# Tn*3-like* structures co-harboring of *bla*_*CTX-M-65*_*, bla*_*TEM-1*_ and *bla*_*OXA-10*_ in the plasmids of two *Escherichia coli* ST1508 strains originating from dairy cattle in China

**DOI:** 10.1186/s12917-023-03847-2

**Published:** 2023-12-18

**Authors:** Weiwei Wang, Xiaojuan Wei, Zhen Zhu, Lingyu Wu, Qiqi Zhu, Safia Arbab, Chengye Wang, Yubin Bai, Qing Wang, Jiyu Zhang

**Affiliations:** 1 Key Laboratory of New Animal Drug Project of Gansu Province, Lanzhou, Gansu Province 730050 People’s Republic of China; 2 Key Laboratory of Veterinary Pharmaceutical Development, Ministry of Agriculture, Lanzhou, Gansu Province 730050 People’s Republic of China; 3 Lanzhou Institute of Husbandry and Pharmaceutical Sciences, Chinese Academy of Agricultural Sciences, Jiangouyan, Qilihe District, Lanzhou, Gansu Province 730050 People’s Republic of China; 4 College of Life Science and Food Engineering, Hebei University of Engineering, Handan, Hebei Province 056038 People’s Republic of China; 5 College of Veterinary Medicines, Gansu Agriculture University, Lanzhou, Gansu Province 730070 People’s Republic of China

**Keywords:** *Bla*_*CTX-M-65*_, Tn*3-like*, Transmission, *E. coli*, Dairy cattle

## Abstract

The purpose of this study was to determine the level of horizontal transmission of the *bla*_*CTX-M-65*_ gene and the role of its associated mobile genetic elements (MGEs) in the bovine-derived *Escherichia coli*. After PCR identification, two plasmids carrying *bla*_*CTX-M-65*_ were successfully transferred to the recipient *E. coli* J53 Azr through conjugation assays and subsequently selected for Whole-Genome sequencing (WGS) analysis. The resistance profiles of these two positive strains and their transconjugants were also determined through antimicrobial susceptibility tests. Whole genome data were acquired using both the PacBio sequencing platform and the Illumina data platform. The annotated results were then submitted to the Genbank database for accession number recording. For comparison, the genetic environment of plasmids carrying the resistance gene *bla*_*CTX-M-65*_ was mapped using the Easyfig software. WGS analysis revealed Tn*3-like* composite transposons bearing *bla*_*CTX-M-65*_, *bla*_*TEM-1*_, and *bla*_*OXA-10*_ in the IncHI2-type plasmids of these two *E. coli* ST1508 strains. A phylogenetic tree was generated from all 48 assembled *E. coli* isolates *bla*_*CTX-M-65*_, *bla*_*TEM-1*_, and *bla*_*OXA-10*_ from the NCBI Pathogen Detection database with our two isolates, showing the relationships and the contribution of SNPs to the diversity between genetic samples. This study suggests that the transmissibility of *bla*_*CTX-M-65*_ on Tn*3-like* composite transposons contributes to an increased risk of its transmission in *E. coli* derived from dairy cattle.

## Introduction

Since the 1980s, extended-spectrum β-lactamases (ESBL)-producing *E. coli* has spread globally, thus becoming the primary cause of nosocomial and community-acquired infections such as gastrointestinal and extra-intestinal infections which majorly includes urinary tract infections (UTIs) [1, 2]. Most of *E. coli* strains are extended beta-lactamase producing and identified as multidrug resistant (resistance to penicillin, cephalosporin’s, and monobactems) due to the hydrolyzaiton of beta-lactam ring. The most prevalent ESBL families include CTX-M, TEM, OXA and SHV, with CTX-M gradually emerging as the dominant variant [3–5]. ​The expression of CTX-M family genes is often associated with increase drug resistance to multiple beta-lactam antibiotics which restrict the more options for therapeutic purposes leading to the treatment options are severely limited [6]. In addition, *CTX-M* genes have been widely identified in *E. coli* isolated from multiple sources such as humans, livestock, companion animals, food products, and environment, raising significant concerns [1, 6].

The *CTX-M* genes are rarely found on chromosomes, instead encoded on mobile genetic elements (MGEs), usually on plasmids. Plasmid located *CTX-M* genes has a strong frequency of horizontal transfer and their increased dissemination within one health care (humans, animals, and environment) [7, 8]. MGEs located on plasmids can widely spread antibiotic resistance genes (ARGs) [9]. It follows that Enterobacteriaceae have adopted a crucial strategy to capture, stabilize and disseminate new genetic elements during the acquisition and evolution of ESBL enzymes [6]. The current epidemiological scenario suggests that the transmission pattern of the CTX-M enzyme may be allodemic rather than epidemic. In other words, the widespread prevalence of CTX-M enzyme is not due to the proliferation of specific clones, but rather to the proliferation of multiple specific clones or MGEs [10].

Co-transmission of *CTX-M-65* with other ESBL-ARGs in *E. coli* have also been reported more frequently, ultimately leading to an increased risk of livestock-to-human transmission and global multidrug resistance (MDR) through horizontal gene transfer (HGT) [11–14]. Note that *bla*_*CTX-M-65*_ gene, with an elevated detection rate, has been reported in food animals [15, 16]. Food-producing chickens carrying extended-spectrum β-lactamase–producing *Escherichia coli* (ESBL-EC), including *CTX-M-65*–producing *Escherichia coli* have posed a potential threat to human and animal health [17]. As a result, there is an increased risk of transmission of these genes from food animals to humans. In this study, we characterized and investigated the horizontal transfer mechanism of *bla*_*CTX-M-65*_ from bovine origin through whole genome sequencing and conjugation assay.

## Materials and methods

### Strain isolation

73 non-repetitive fecal samples were collected from dairy cattle in the Xinjiang region of China in 2018. All samples were collected in sterile containers, transported to the laboratory at 6 °C ± 2 °C, and processed immediately for further assays. All isolates were selected on MacConkey agar plates (Huankai, China) and identified by MALDI-TOF–MS and 16 s rRNA sequencing using universal primers F (5’-GAGCGGATAACAATTTCACACAGG-3’) and R (5’-CGCCAGGGTTTTCCCAGTCACGAC-3’). All *E. coli* strains were stored in our laboratory after identification.

*CTX-M*-65-positive strains were screened for the presence of *bla*_*CTX-M-9*_ group using PCR methods with primers obtained from the article [18]. PCR products were confirmed using Sanger sequencing.

### Conjugation assay and determination of conjugation frequency

Conjugation assays were performed among 2 isolates. To determine the transferability of the resistance genes, filtered broth mating was performed using *E. coli* J53 Az^r^ as the recipient [19]. The donor-recipient ratio was 1:1 using Mueller–Hinton medium (MHA, Huankai, Guangzhou, China) supplemented with cefotaxime (2 μg/mL) and sodium azide (200 μg/mL) as the selective medium. Positive clones of transconjugants were screened by PCR (*bla*_*CTX-M-9*_ primers, F: 5’-ATGGTGACA AAG AGAGTGCAAC-3’, R: 5’-TTACAGCCCTTCGGCGATG-3’), Sanger sequencing and antimicrobial susceptibility testing. Transfer frequencies were calculated as the number of transconjugants per recipient.

### Antimicrobial susceptibility testing and resistant genes detecting

Antimicrobial susceptibility testing was performed using the Kirby-Bauer disk diffusion method according to Clinical & Laboratory Standards Institute (CLSI) M100 guidelines (http://clsi.org). Broth culture, equivalent to a 0.5 McFarland standard, was incubation for 16 h at 37℃. The following antibiotics were used for testing: cefotaxime (CTX), ceftazidime (CAZ), cefalotin (KF), tetracycline (TE), ampicillin (AMP), sulbactam-ampicillin (SAM), streptomycin (S), amoxicillin/clavulanic acid (AMC), amikacin (AK), ciprofloxacin (CIP), doxycycline (DO), fosfomycin (FOT), kanamycin (K), chloramphenicol (C), sulphamethoxazole-trimethoprim (SXT), gentamicin (GN), aztreonam (ATM). *E. coli* ATCC25922 was used as the quality control strain.

### WGS sequencing and analysis

The total genomic DNA was extracted with the SDS method [20]. The harvested DNA was detected by the agarose gel electrophoresis and quantified by Qubit® 2.0 Fluorometer (Thermo Scientific). The whole genome of the isolate was sequenced on PacBio Sequel single-molecule real-time (SMRT) sequencing platform (Novogene Bioinformatics Technology Co., Beijing, China) with 400 bp paired-end libraries. It was preliminarily assembled with SMRT Link v5.0.1. The assembled data were optimized by Illumina reads using Burrows-Wheeler Aligner (BWA).

Gene prediction and annotation were performed using the RAST server (https://rast.nmpdr.org/) and the BLAST program of NCBI (https://blast.ncbi.nlm.nih.gov/Blast.cgi). The replicon of the plasmid was confirmed using PlasmidFinder 2.1 (with minimum identity 95% and minimum coverage 60%) (https://cge.cbs.dtu.dk/services/PlasmidFinder/). The antibiotic-resistant genes were identified using CARD data (https://card.mcmaster.ca/). Clonal analysis was assessed by MLST 2.0 (https://cge.food.dtu.dk/services/MLST/). The comparative analysis and plasmid map was generated with Easyfig2.2.3 [21]. The pathogenic and antibiotic-resistant information of strainswas supported by NCBI Pathogenic Detection (https://www.ncbi.nlm.nih.gov/pathogens/).

To confirm the phylogenetic relationship based on the single nucleotide polymorphisms (SNPs) present in the genomes carrying the *bla*_*CTX-M-65*_ gene, the two *E. coli* strains in this study and the 48 *E. coli* strains downloaded from the NCBI Pathogenic Detection were analyzed using CSI Phylogeny version 1.4 (10 reads of minimal depth at SNP positions, 10% minimal relative depth at SNP positions, 10 bp of minimal distance between SNPs, minimal SNP quality of 30, minimal read mapping quality of 25, and a minimal Z-score of 1.96 [22]. The phylogenetic tree was visualized using FigTree version v1.4.4.

### Phylogenetic analysis

The phylogenetic analysis was performed using the method of Kaas [23]. A phylogenetic tree was generated using *E. coli* ATCC25922 as the reference genome for all assembled *E. coli* isolates carrying *bla*_*CTX-M-65*_, *bla*_*TEM-1*_, and *bla*_*OXA-10*_ from the NCBI Pathogenic Detection database. To be specific, a total of 50 *E. coli* isolates carrying these ARGs were collected from different regions of the world and isolated from various sources.

### Nucleotide sequence accession number

The data of the whole-genome sequence was uploaded and registered in the NCBI database with accession numbers: CP098227 (*E. coli* XJ6.3-chr1), CP098228 (*E. coli* XJ6.3-plas1), CP098231 (*E. coli* XJ34-chr1), CP098232 (*E. coli* XJ34-plas1), CP098233 (*E. coli* XJ34-plas2).

### Availability of data and materials

The datasets generated and analyzed during the current study are available in the [NCBI] repository with accession numbers as mentioned above.

## Results and discussion

A total of 108 *E. coli* strains were isolated on MacConkey agar from dairy cow and confirmed by 16 s rRNA and MALDI-TOF–MS. Among the 108 *E. coli* strains, three strains were identified positive for *bla*_*CTX-M-65*_ gene by PCR method in our previous study [24]. In two strains, *bla*_*CTX-M-65*_ gene was successfully transferred through conjugation which were further characterized for antimicrobial susceptibility testing, acquired ARGs, and related the genetic environment through WGS analysis. The information about the origin and source of the isolates is shown in Table 1.

**Table 1:** Background information and characteristics of *bla*_*CTX-M-65*_-positive isolates

Strains	Location	Source	Origin	MLST	Serotype	Resistance Phenotype
XJ6.3	Xinjiang Province	Dairy Cattle	Fecal samples	ST1508	O172:H45	KF/CTX/CAZ/TE/C/AMP/DO/ATM/SAM/SXT
XJ34	Xinjiang Province	Dairy Cattle	Fecal samples	ST1508	O172:H45	KF/CTX/CAZ/TE/C/AMP/DO/SAM/SXT

*CTX* cefotaxime, *CAZ* ceftazidime, *KF* cefalotin, *TE* tetracycline, *AMP* ampicillin, *SAM* sulbactam-ampicillin, *DO* doxycycline, *C* chloramphenicol, *SXT* sulphamethoxazole-trimethoprim, *ATM* aztreonam

The Kirby-Bauer disk diffusion method was used to evaluate the drug resistance profile against multiple antibiotics. The results showed that both of the strains were multidrug-resistant (MDR) and resistant to at least nine antibiotics. Both of the isolates showed resistance to cefalotin (KF), cefotaxime (CTX), tetracycline (TE), chloramphenicol (C), ampicillin (AMP), doxycycline (DO), sulbactam-ampicillin (SAM) and strimethoprim-sulfamethoxazole (SXT) (Table 1). A previous conducted by researchers also identified MDR *E. coli* strains isolated from dairy origin which is consistent to the current findings [25]. The ESBL-producing *E. coli* also displayed a wide spectrum antibiotic resistance to other commonly used antibiotics at clinical and livestock industry such as β-lactam, tetracycline and chloramphenicol and trimethoprim-sulfamethoxazole which results in limited treatment options and higher rate of therapeutic failures in infections caused by ESBL-producing *E. coli*.

The conjugation assay revealed that two plasmids carrying *CTX-M-65* gene were successfully transferred to the recipient *E. coli* J53 (Sodium Azide resistance) indicating the presence of the target gene on a mobile plasmid. The conjugation frequencies of XJ6-3-J53 and XJ34-J53 carrying *CTX-M-65* gene were noted to be 1.9 × 10^–6^ and 6.9 × 10^–5^ conjugants per recipient respectively which is in consistent with a previous study [26].

Both of the strains were sent for WGS and characterized for plasmid replicon typing, serotyping, MLST, acquired ARGs, and phylogenetic relationship using CGE website. Plasmid replicon typing revealed that *bla*_*CTX-M-65*_ gene was localized on IncHI2 plasmid type in both strains along with other replicon types such as IncI1, IncF, and IncA/C2 plasmids (Table 1) which are previously known as dominant plasmid replicon types [11, 27].

The MLST revealed that both *CTX-M-65*-positive strains belongs to ST1508. Surprisingly, after searching the Pathogen Detection database, none of the *CTX-M-65* positive strain was ST1508 which indicate the emergence of a novel ST1508 in this study. Moreover, both XJ6.3 and XJ34 strains were categorized under O172:H45 serotype which belongs to toxin producing *E. coli*. *E. coli* O172 producing Shiga toxin type 2 has been reported from food and water sources commonly associated with illnesses [28, 29]. XJ6.3 and XJ34 have certain degree of pathogenicity and resistance, therefore their possible transmission need greater attention at the right time.

​Two plasmids carrying *CTX-M-65* gene were successfully transferred to the recipient *E. coli* J53 Azr according to conjugation assays, indicating the presence of the *CTX-M-65* gene on a plasmid. The conjugation frequencies of J53XJ6-3 and J53XJ34 carrying *CTX-M-65* gene were 1.9 × 10^–6^ and 6.9 × 10^–5^, respectively. The transconjugative frequencies of conjugants of harboring *CTX-M-65* genes were 10^–5^ ~ 10^–6^ in consistent with the additional research of *CTX-M-65* on IncHI2 [26].

**Fig. 1:**
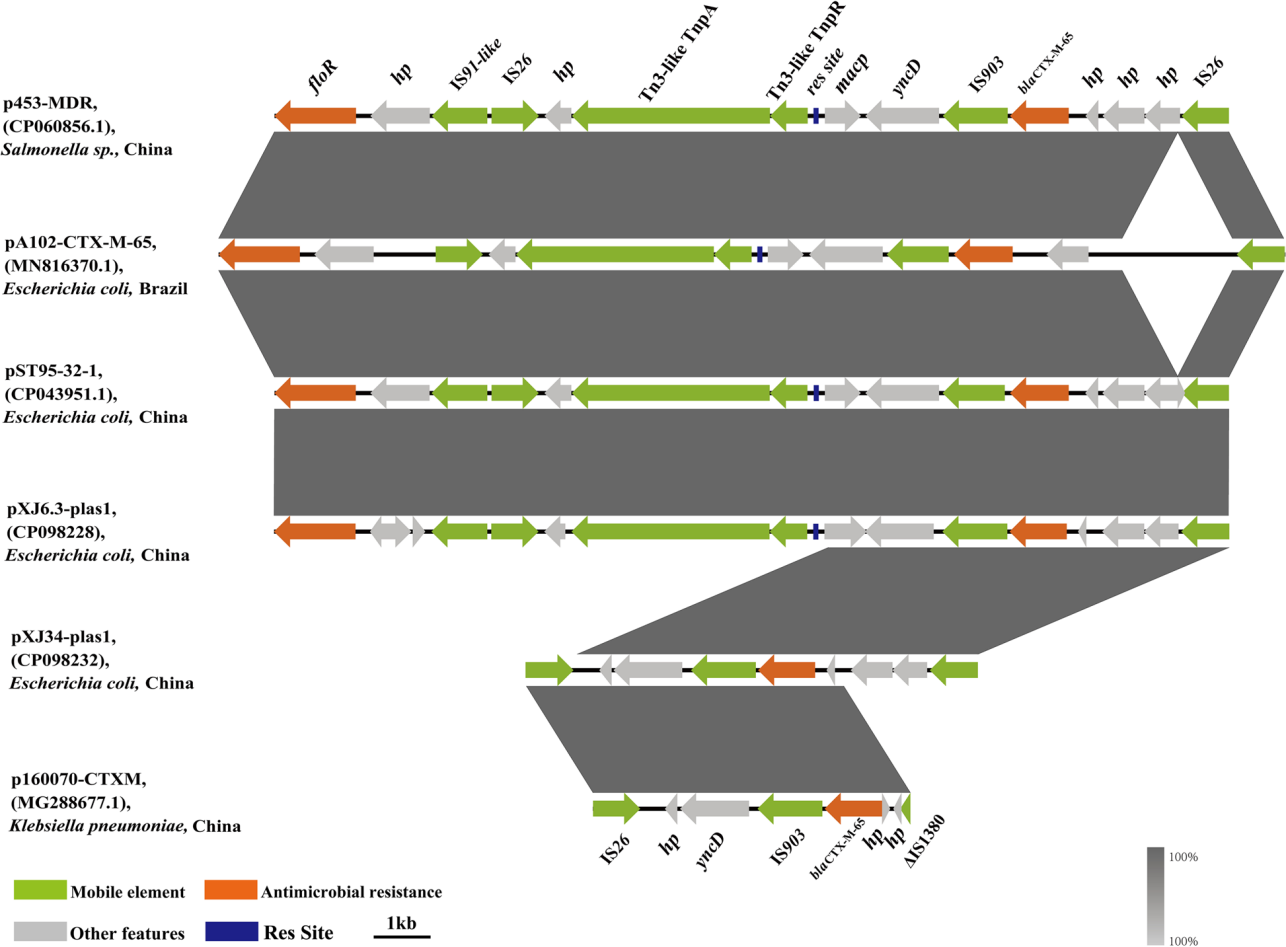
Sequence alignment of *bla*_*CTX-M-65*-_positive IncHI2 plasmids of pXJ6.3-plas1 and pXJ34-plas1

The genetic environment analysis of two *bla*_*CTX-M-65*_-positive isolates were done by WGS. For pXJ6.3-plas1, the complete genome contained a circular 181,896 bp plasmid with GC content of 46.0%, harboring *bla*_*CTX-M-65*_, *bla*_*TEM-1*_, *tet(A)*, *floR*, *dfrA14*, *aadA1*, *bla*_*OXA-10*_, *cmlA5*, *arr-2*, and *qnrS1* genes (Table 2). This plasmid was also harboring several mobile elements, including IS*91*-like, Tn*3-like*, IS*26*, IS*2*, IS*6* and Int*1*. The genetic region of 14,251 bp around the *bla*_*CTX-M-65*_ gene was found 99.8% ~ 99.9% identical with 100% coverage to three plasmids, pA102-*CTX-M-65* carried by *E. coli* (MN816370.1), p453-MDR carried by *Salmonella sp.* (CP060856.1) and pST95-32–1 carried by *E. coli* (CP043951.1), respectively (Fig. 1). The genome of *E. coli* XJ34 was carrying a circular 185,577-bp with GC content of 46.0%. The *bla*_*CTX-M-65*_ positive isolates harboring IncHI2 plasmid were acquiring same resistance genes with two additional mobile elements, IS*903* and ΔIS*1380* (Table 2). The genomic region of 6,747 bp was found 99.8% similar in sequence identity (coverage 100%) to the plasmid, p160070-CTXM carried by *Klebsiella pneumonia* (MG288677.1.1) (Fig. 1). These observation suggested that these multidrug-resistant region (MRR) on IncHI2-type plasmids carrying *bla*_*CTX-M-65*_ have a high degree of similarity, indicating Tn*3-like*–1S903-*bla*_*CTX-M-65*_–IS*26* were conducive to spread between different species.

**Table 2:** Characterization of plasmids carrying *bla*_*CTX-M-65*_

Plasmids	Size(Kb)	Replicon type	MGEs	Resistance genes
XJ6.3-plas1	180	IncHI2	IS*91, Tn3, *IS*26, *IS*2, *IS*6, Int1*	*bla*_*CTX-M-65*_/*bla*_*TEM-1*_/*tet(A)*/ *floR/dfrA14/aadA1/bla*_*OXA-10*_*/cmlA5/arr-2/qnrS1*
XJ34-plas1	180	IncHI2	IS*903, *IS*1380, Tn3, *IS*26, *IS*2, *IS*6, Int1*	*bla*_*CTX-M-65*_/*bla*_*TEM-1*_*/tet(A)*/ *floR/dfrA14*/*aadA1*/*bla*_*OXA-10*_/*cmlA5/arr-2/qnrS1*

The results of genetic context showed that both strains were also carrying additional beta-lactam resistance genes, *bla*_*TEM-1*_ and *bla*_*OXA-10*_. More than 170 *TEM-1* variants have been identified in *E. coli* from hospitals and clinical settings worldwide, which can hydrolyze beta-lactam drugs such as penicillin and cephalosporin’s [30]. The OXA family enzymes are mostly limited to penicillin group, but recently their resistance to last resort carbapenem was also observed which needs much attention because of horizontal transfer of genes as observed in this study *in-vitro* trial.

The mobile genetic elements such as IS*903* and Tn*3* are the most prevalent insertions and transposons which associated with *bla*_*CTX-M-65*_ which is consistent with our findings that *bla*_*CTX-M-65*_ gene was surrounded by IS*903* and Tn*3-like* mobile elements [31]. The Tn*3* belong to bacterial transposons group which are involved in transposition function and mediation of gene re-assortment, which make it conducive for horizontal gene transfer in gram-positive and negative bacteria [32]. In addition, the res site provides more clarity on the Tn*3-like* transposon formed by transduction and integration to transmit ARGs. The presence of these elements in the genome of *bla*_*CTX-M-65*_*-*positive strains mediates the mechanism of genes mobilization and increase the risk of horizontal transmission through multiple mobile elements [33].

**Fig. 2:**
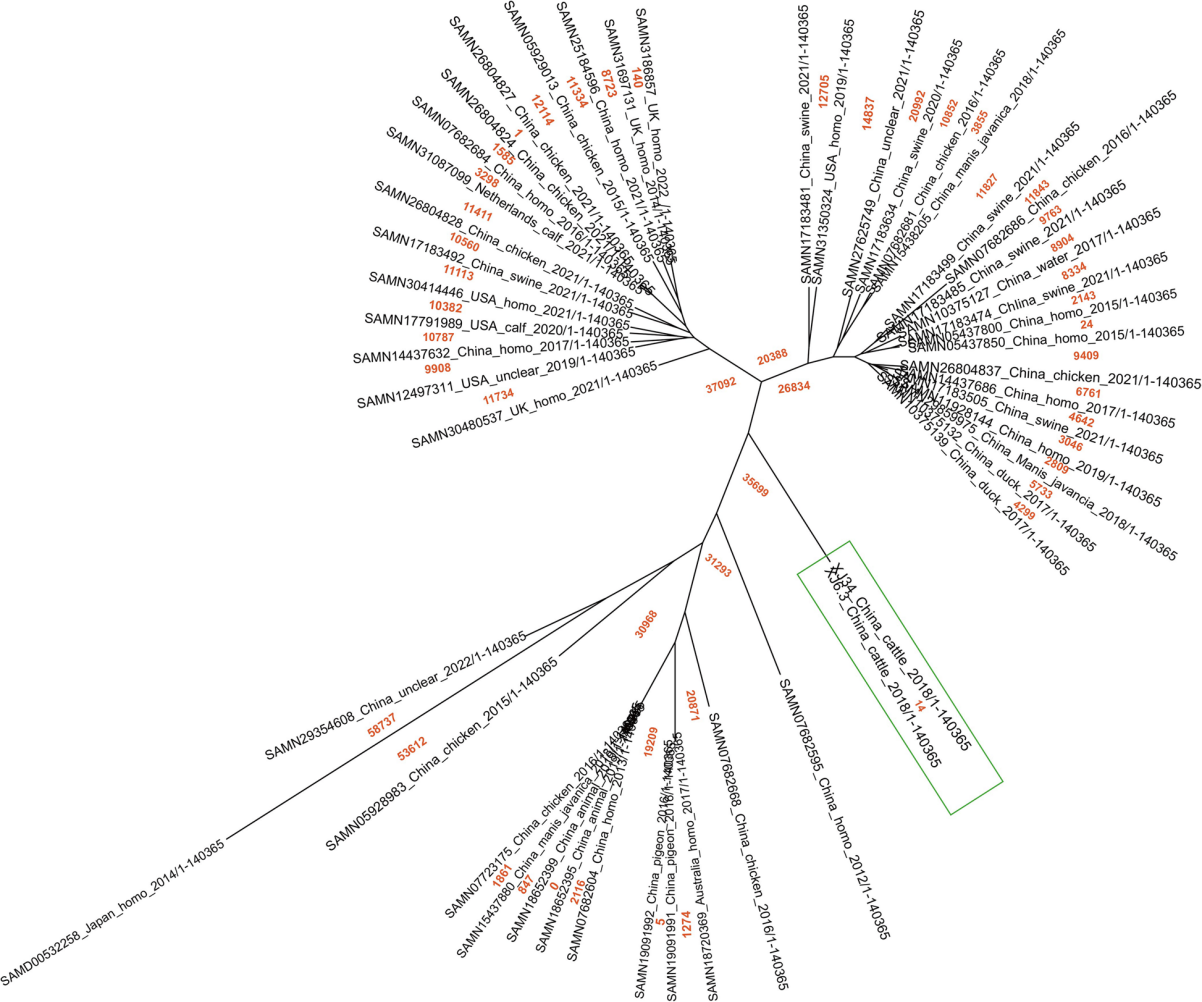
Phylogenetic trees from SNP analysis of 50 *E. coli* strains carrying the *bla*_*CTX-M-65*_, *bla*_*TEM-1*_, and *bla*_*OXA-10*_ genes. The orange Arabic numerals indicate the number of SNPs for different strains from various regions and sources. Isolated strains in this study are marked with a green box

The phylogenetic relationship of two strains harboring *bla*_*CTX-M-65*_ gene and other strains retrieved from NCBI database were done by CSIPhylogeny tool based on SNPs. Both strains of present study shared 14 SNPs with each other indicating much homology with each other. However, both strains shared lower homology with other 48 strains retrieved from NCBI based on SNP differences ranging from 26,834 to 35,699. In addition, SNP analysis showed that SAMD00532258 strain were having highest SNPs, between 58,737 and 53,612. The number of SNPs in the majority of the strains carrying three ARGs were ranged from 847 to 58,737. The phylogenetic tree distinguished the two strains of this study and 48 from NCBI into 4 major clades (Fig. 2). Clade 1 consist of 20 strains, one from USA and left from China isolated from chicken, swine, duck, *Manis javancia*, human and water sources. Clade 2 were containing 15 strains from four different countries and sources. Clade 3 were consisting of two strains characterized in present study harboring *bla*_*CTX-M-65*_ under separate sub-clade and 13 other strains isolated from humans and animals in China. The phylogenetic analysis revealed that there was a high degree of genetic homology between the two strains (XJ6.3 and XJ34), marked with green box of this study, and lower homology with other strains. The aforementioned genetic differences from phylogenetic analysis indicate that these genes are more likely to be transmitted horizontally by plasmids rather than clonal dissemination.

## Conclusion

The WGS analysis obtained a complete map of the genetic environment of *CTX-M-65* in both isolates through the Pacbio sequencing platform and Illumina data platform. The *CTX-M-65* positive *E. coli* isolates derived from dairy cattle and was to show co-transmission of *bla*_*CTX-M-65*_ with other ESBLs genes (*bla*_*TEM-1*_, *bla*_*OXA-10*_) on the IncHI2 plasmid. A genetic environmental analysis of the *CTX-M-65* gene revealed the presence of Tn*3-like* transposons and IS*903* in both strains, possibly contributing to the mobilization of those ARGs. Genetic differences from phylogenetic analysis display showing the relationships and the contribution of SNPs to the diversity between genetic samples and co-transmission of these genes is more likely to occur through plasmids than through clones. Also, *CTX-M-65* was derived from normal dairy cattle in this study, and it is still not absolutely certain that the source of *CTX-M-65* is food dairy cattle. Finally, this study provides evidence of the need for appropriate measures to minimize the spread of MDR bacteria in dairy cattle samples.

## Data Availability

The datasets generated and analyzed during the current study are available in the [NCBI] repository with accession numbers: CP098227 (*E. coli* XJ6.3-chr1), CP098228 (*E. coli* XJ6.3-plas1), CP098231 (*E. coli* XJ34-chr1), CP 098232 (*E. coli* XJ34-plas1), CP 098233 (*E. coli* XJ34-plas2).

## Abbreviations

MGEs: Mobile genetic elements
*E. coli*: *Escherichia coli*
WGS: Whole-Genome sequencing
ESBL: Extended-spectrum β-lactamases
ARGs: Antibiotic resistance genes
MDR: Multidrug resistance
CTX: Cefotaxime
CAZ: Ceftazidime
KF: Cefalotin
TE: Tetracycline
AMP: Ampicillin
SAM: Sulbactam-ampicillin
S: Streptomycin
AMC: Amoxicillin/clavulanic acid
AK: Amikacin
CIP: Ciprofloxacin
DO: Doxycycline
FOT: Fosfomycin
K: Kanamycin
C: Chloramphenicol
SXT: Sulphamethoxazole-trimethoprim
GN: Gentamicin
ATM: Aztreonam
SMRT: Single-molecule real-time
ICE: Integrative and conjugative element
ST: Sequence type
SNPs: Single nucleotide polymorphisms
